# Supplementary figures and images for: Prediction of hypertension using traditional regression and machine learning models: A systematic review and meta-analysis
Source: PLoS One. 2022 Apr 7;17(4):e0266334. doi: 10.1371/journal.pone.0266334 (PMC8989291; doi:10.1371/journal.pone.0266334)

**S1 Fig.** The number of PROBAST criteria satisfied by different studies.


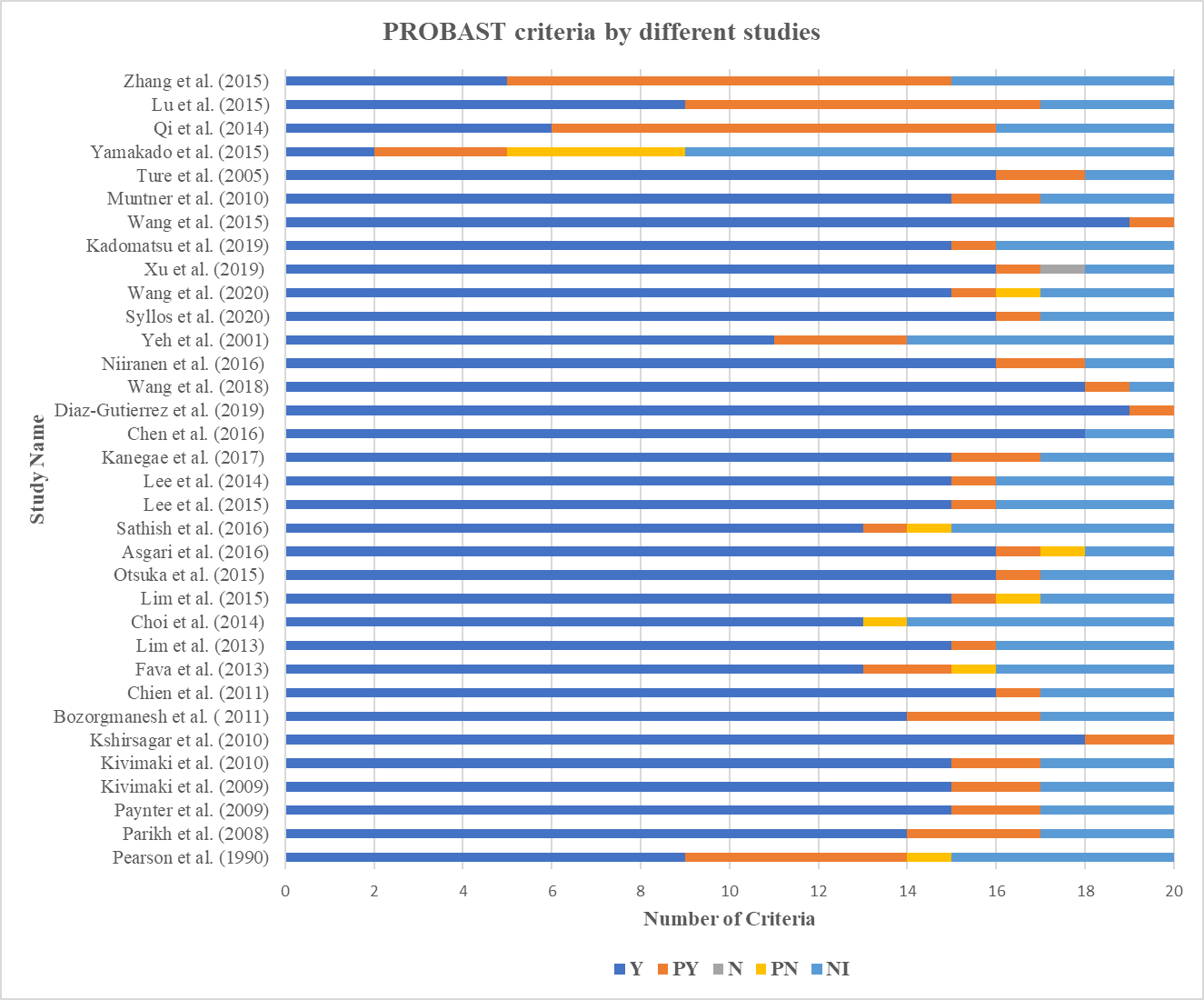

Supplement: S1 Fig — (DOC) [file pone.0266334.s002.DOC]

**S2 Fig.** Response to different signaling questions by the number of studies.


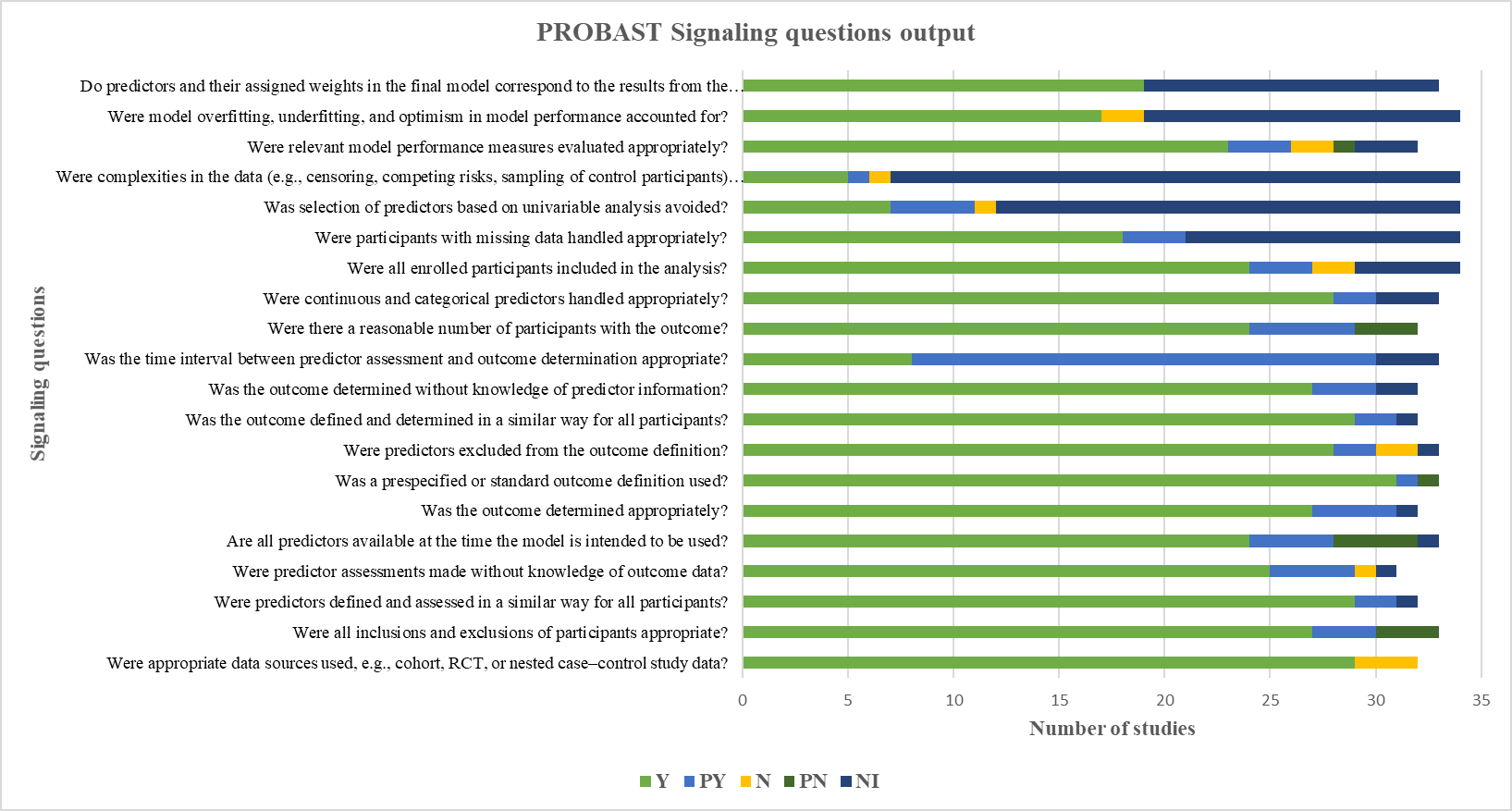

Supplement: S2 Fig — (DOC) [file pone.0266334.s003.DOC]

**S3 Fig.** Forest plot of externally validated models with 95% prediction interval.

**
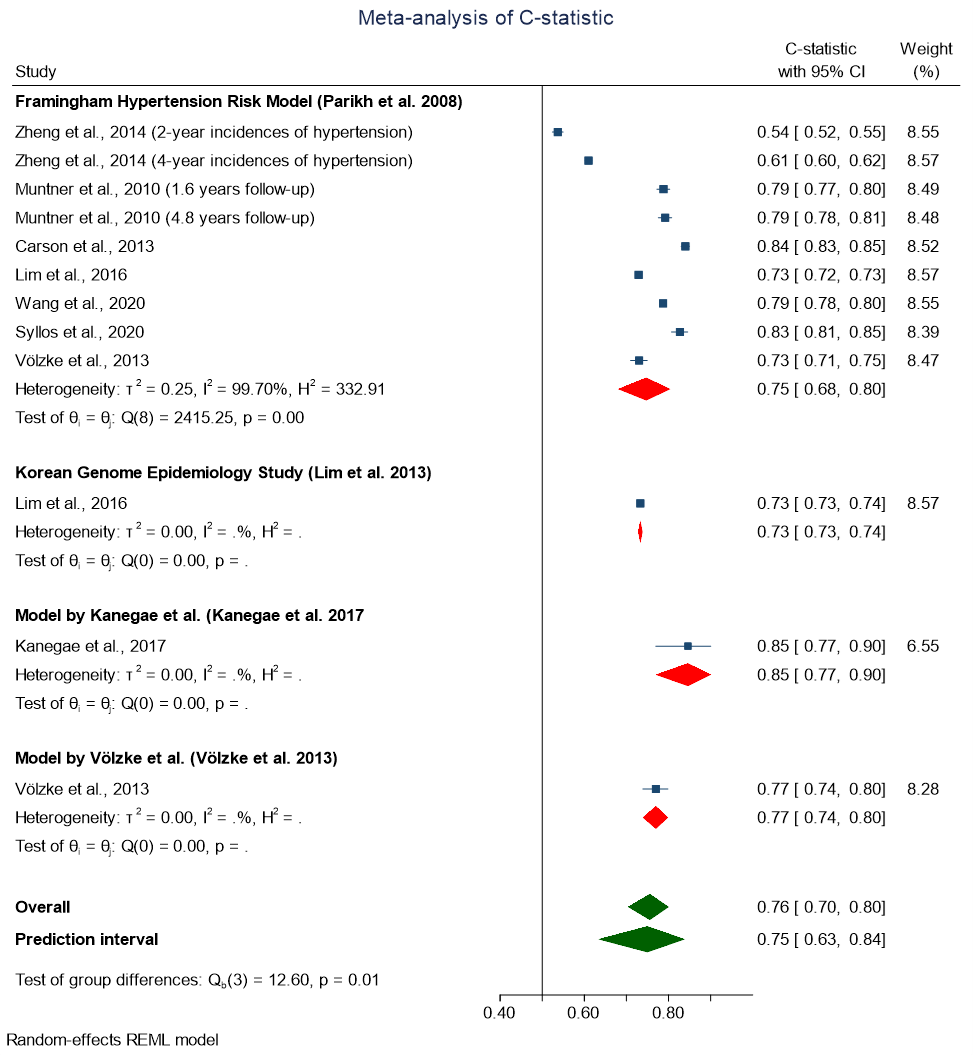
**

Supplement: S3 Fig — (DOC) [file pone.0266334.s004.DOC]
