# Supplementary material for: Prediction of hypertension using traditional regression and machine learning models: A systematic review and meta-analysis
Source: PLoS One. 2022 Apr 7;17(4):e0266334. doi: 10.1371/journal.pone.0266334 (PMC8989291; doi:10.1371/journal.pone.0266334)

**S4 Fig.** Forest plot of models primarily developed using genetic risk factors/biomarkers with a 95% prediction interval.


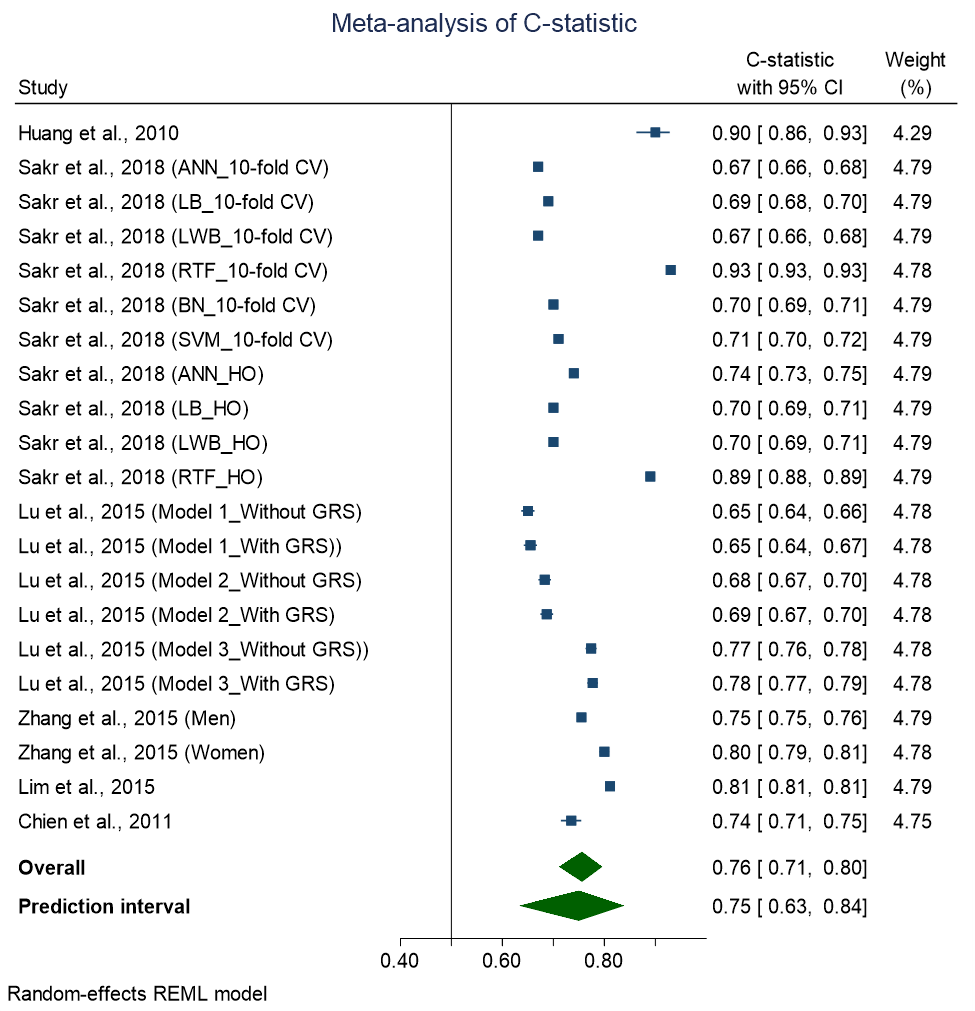

Supplement: S4 Fig — (DOC) [file pone.0266334.s005.DOC]
