# Supplementary material for: Prediction of hypertension using traditional regression and machine learning models: A systematic review and meta-analysis
Source: PLoS One. 2022 Apr 7;17(4):e0266334. doi: 10.1371/journal.pone.0266334 (PMC8989291; doi:10.1371/journal.pone.0266334)
Supplement: S1 Table — (DOC) [file pone.0266334.s006.DOC]

**S1 Table.** Keywords Used to Search in MEDLINE

| **KEYWORDS** |
| --- |
| 1. prediction model*.mp 2. risk function*.mp 3. risk prediction*.mp 4. risk table*.mp 5. predictive model*.mp 6. exp "Predictive Value of Tests"/ 7. risk chart*.mp 8. risk equation*.mp 9. risk engine*.mp 10. risk calculat*.mp 11. risk score*.mp 12. prediction tool*.mp 13. prediction rule*.mp 14. risk model*.mp 15. prognostic tool*.mp 16. prognostic model*.mp 17. exp Risk Assessment/ 18. risk algorithm*.mp 19. risk ind*.mp 20. prediction algorithm*.mp 21. (hypertension adj2 (risk score or risk model or prediction model or risk prediction model or risk assessment)).mp 22. (high blood pressure adj2 (risk score or risk model or prediction model or risk prediction model or risk assessment)).mp 23. OR/1-22 24. validation.mp. 25. exp Validation Studies/ 26. validate*.mp 27. OR/24-26 28. 23 AND 27 29. exp Hypertension/ 30. hypertens*.mp 31. (high adj2 blood pressure).mp 32. high blood pressure.mp 33. elevated blood pressure.mp 34. blood pressure.mp 35. OR/29-34 36. 28 AND 35 |
