# Supplementary material for: Prediction of hypertension using traditional regression and machine learning models: A systematic review and meta-analysis
Source: PLoS One. 2022 Apr 7;17(4):e0266334. doi: 10.1371/journal.pone.0266334 (PMC8989291; doi:10.1371/journal.pone.0266334)
Supplement: S2 Table — (DOC) [file pone.0266334.s007.DOC]

**S2 Table.** Study quality assessment using PROBAST

| Study | Risk of Bias (ROB) | | | | Applicability | | | Overall | |
| --- | --- | --- | --- | --- | --- | --- | --- | --- | --- |
|  | **Participants** | **Predictors** | **Outcome** | **Analysis** | **Participants** | **Predictors** | **Outcome** | **ROB** | **Applicability** |
| Pearson et al.[41] (1990) | **-** | **+** | **+** | **?** | **-** | **+** | **+** | **-** | **-** |
| Parikh et al.[22] (2008) | **+** | **+** | **+** | **+** | **-** | **+** | **+** | **+** | **-** |
| Paynter et al.[42] (2009) | **+** | **+** | **+** | **+** | **-** | **+** | **+** | **+** | **-** |
| Kivimaki et al.[43] (2009) | **+** | **+** | **+** | **+** | **-** | **+** | **+** | **+** | **-** |
| Kivimaki et al.[44] (2010) | **+** | **+** | **+** | **+** | **-** | **+** | **+** | **+** | **-** |
| Kshirsagar et al.[45] (2010) | **+** | **+** | **+** | **+** | **+** | **+** | **+** | **+** | **+** |
| Bozorgmanesh et al.[25] ( 2011) | **+** | **+** | **+** | **+** | **+** | **+** | **+** | **+** | **+** |
| Chien et al.[24] (2011) | **+** | **+** | **+** | **+** | **+** | **+** | **+** | **+** | **+** |
| Fava et al.[46] (2013) | **+** | **+** | **+** | **?** | **+** | **+** | **-** | **?** | **-** |
| Lim et al.[30] (2013) | **+** | **+** | **+** | **?** | **+** | **+** | **+** | **?** | **+** |
| Choi et al.[47] (2014) | **+** | **+** | **+** | **?** | **?** | **-** | **+** | **?** | **-** |
| Lim et al.[48] (2015) | **+** | **+** | **+** | **+** | **+** | **-** | **+** | **+** | **-** |
| Otsuka et al.[23] (2015) | **+** | **+** | **+** | **?** | **-** | **+** | **+** | **?** | **-** |
| Asgari et al.[49] (2016) | **+** | **-** | **+** | **+** | **+** | **+** | **-** | **-** | **-** |
| Sathish et al.[29] (2016) | **+** | **+** | **+** | **-** | **+** | **+** | **+** | **-** | **+** |
| Lee et al.[50] (2015) | **+** | **+** | **+** | **?** | **+** | **+** | **+** | **?** | **+** |
| Lee et al.[51] (2014) | **+** | **+** | **+** | **+** | **+** | **-** | **+** | **+** | **-** |
| Kanegae et al.[32] (2017) | **+** | **+** | **+** | **+** | **-** | **+** | **+** | **+** | **-** |
| Chen et al.[52] (2016) | **+** | **+** | **+** | **+** | **+** | **+** | **+** | **+** | **+** |
| Diaz-Gutierrez et al.[28] (2019) | **+** | **+** | **+** | **+** | **-** | **+** | **+** | **+** | **-** |
| Wang et al.[53] (2018) | **+** | **+** | **+** | **+** | **+** | **+** | **-** | **+** | **-** |
| Niiranen et al.[54] (2016) | **+** | **+** | **+** | **+** | **+** | **-** | **+** | **+** | **-** |
| Yeh et al.[55] (2001) | **+** | **+** | **+** | **?** | **+** | **-** | **-** | **?** | **-** |
| Syllos et al.[21] (2020) | **+** | **+** | **+** | **+** | **+** | **+** | **+** | **+** | **+** |
| Wang et al.[27] (2020) | **+** | **+** | **+** | **+** | **+** | **+** | **+** | **+** | **+** |
| Xu et al.[56] (2019) | **+** | **+** | **+** | **-** | **+** | **+** | **+** | **-** | **-** |
| Kadomatsu et al.[26] (2019) | **+** | **+** | **+** | **?** | **+** | **+** | **+** | **?** | **+** |
| Wang et al.[57] (2015) | **+** | **+** | **+** | **+** | **+** | **+** | **+** | **+** | **+** |
| Muntner et al.[58] (2010) | **+** | **+** | **+** | **+** | **+** | **+** | **+** | **+** | **+** |
| Ture et al.[59] (2005) | **+** | **+** | **+** | **+** | **?** | **+** | **+** | **+** | **?** |
| Yamakado et al.[60] (2015) | **-** | **-** | **-** | **-** | **+** | **-** | **-** | **-** | **-** |
| Qi et al.[61] (2014) | **+** | **+** | **+** | **?** | **-** | **-** | **-** | **?** | **-** |
| Lu et al.[62] (2015) | **+** | **+** | **+** | **?** | **+** | **-** | **+** | **?** | **-** |
| Zhang et al.[63] (2015) | **+** | **+** | **+** | **?** | **+** | **-** | **+** | **?** | **-** |
