# Supplementary material for: Prediction of hypertension using traditional regression and machine learning models: A systematic review and meta-analysis
Source: PLoS One. 2022 Apr 7;17(4):e0266334. doi: 10.1371/journal.pone.0266334 (PMC8989291; doi:10.1371/journal.pone.0266334)
Supplement: S3 Table — (DOC) [file pone.0266334.s008.DOC]

**S3 Table.** Information about external validation studies of existing traditional hypertension prediction models from selected studies

| **Study Name/Prediction Model Validated** | **Total Number of Validation Studies** | **Validation Study** | **Location/Ethnicity** | **Age** | **Follow-up Period** | **Events (n)/Total Participants (N)** | **Outcome Definition** | **Calibration** | **Discrimination** |
| --- | --- | --- | --- | --- | --- | --- | --- | --- | --- |
| Parikh et al.[22] 2008/Framingham Hypertension Risk Model (FHRS) | 8 | Zheng et al.[75] 2014 | China/Asians | ≥ 35 years | Median 4.8 years | 8675/24,434 | Average SBP ≥140 mm Hg, and/or DBP ≥ 90 mm Hg, and/or use of antihypertensive medications within 2 weeks before the follow-up examination | H–L Chi-square test = 2,287.7 (P < 0.0001), 2-year incidence of hypertension H–L Chi-square test = 8,227.1 (P < 0.0001), 4-year incidence of hypertension | C statistics = 0.537 [0 .524–0.550], 2-year incidences of hypertension  C statistics = 0.610 [0.602–0.618], 4-year incidences of hypertension |
|  |  | Muntner et al.[58] 2010 | USA/Multiethnic (Whites, Blacks, Hispanics, and Asians–primarily of Chinese descent) | 45-84 years | Median of 1.6 years and 4.8 years | 849/3013 | The first study visit, subsequent to baseline, at which SBP ≥ 140 mm Hg and/or DBP ≥ 90 mm Hg and/or the initiation of antihypertensive medication | H-L goodness of fit Chi-square: p < 0.001 | C-statistic = 0.788 [0.773 - 0.804] (1.6 years follow-up)  C-statistic = 0.792 [0.775-0.807] (4.8 years follow-up) |
|  |  | Carson et al.[76] 2013 | USA/Whites and Blacks | 18-30 years | 25 years | 1179/4388 | First study examination in which SBP ≥ 140 mm Hg or DBP ≥ 90 mm Hg or initiated treatment with antihypertensive medications | Modified H–L goodness of fit χ2 = 249.4; P < 0.001 | C-index = 0.84 [0.83–0.85] |
|  |  | Lim et al.[77] 2016 | Korea/Asians | 40–69 years | 4 years | 13005/69,918 | SBP ≥ 140 mmHg or DBP ≥ 90 mmHg on health examination or a record with hypertensive disease codes (I10–I13) and prescription of one of the antihypertensive agents | H-L Chi-square p < 0.001 | AROC = 0.729 |
|  |  | Kivimäki et al.[43] 2009 | England/Mainly Whites | 35-68 years | Median 5.6 years | NR/5472 | SBP ≥ 140 mmHg or DBP ≥ 90 mmHg or use of blood pressure-lowering medications | H-L Chi-square = 11.5 | C-statistic = 0.803 |
|  |  | Wang et al.[27] 2020 | China/Asians | ≥ 18 years | Median 6 years | 1658/9034 | SBP ≥ 140 mm Hg, DBP ≥ 90 mm Hg, or the use of blood pressure-lowering medications | NR | AUC = 0.787 [0.778–0.795] |
|  |  | Syllos et al.[21] 2020 | Brazil/South Americans | 35-74 years | 4 years | 1088/8027; Derivation: 4825 Validation: 3202 | SBP ≥ 140 mm Hg, DBP ≥ 90 mm Hg, or the use of blood pressure-lowering medications | H-L Chi-square = 3.78, p = 0.876 | AUC = 0.827 [0.808 - 0.847] |
|  |  | Völzke et al.[31] 2013 | Denmark/Whites | 20-79 years | 5.4 ± 0.2 years | 434/2887 | SBP ≥ 140 mmHg and DBP ≥ 90 mmHg | Validation dataset: H-L Chi-square = 11.26 (p = 0.19) External validation dataset: H-L Chi-square = 203.34 (p < 0.001) | Validation dataset: AUC = 0.77 [0.73 – 0.82]  External validation dataset: AUC = 0.73 [0.71-0.75] |
| Lim et al.[30] 2013/ Korean Genome Epidemiology Study (KoGES) | 1 | Lim et al.[77] 2016 | Korea/Asians | 40–69 years | 4-year | 13,005/69,918 | SBP ≥ 140 mmHg or DBP ≥ 90 mmHg on health examination, or a record with hypertensive disease codes (I10–I13) and prescription of one of the antihypertensive agents | H-L Chi-square p = 0.062 | AROC = 0.733 |
| Völzke et al.[31] 2013 | 1 | Völzke et al.[31] 2013 | Denmark/Whites | 20-79 years | 5.4 ± 0.2 years | 434/2887 | SBP ≥ 140 mmHg and DBP ≥ 90 mmHg | H-L Chi-square = 40.6 (p < 0.001) | AUC = 0.77 [0.74 – 0.80] |
| Kanegae et al.[32] 2017 | 1 | Kanegae et al.[32] 2017 | Japan/Asians | 18-89 years | Mean 2.4 years | NR/14,168 | SBP/DBP ≥ 140/90 mm Hg and/or the initiation of antihypertensive medications with self-reported hypertension | Greenwood-Nam-D’Agostino χ2 statistic = 8.7 | C-statistic = 0.846 [0.775-0.905] |
