# Supplementary material for: Prediction of hypertension using traditional regression and machine learning models: A systematic review and meta-analysis
Source: PLoS One. 2022 Apr 7;17(4):e0266334. doi: 10.1371/journal.pone.0266334 (PMC8989291; doi:10.1371/journal.pone.0266334)
Supplement: S4 Table — (DOC) [file pone.0266334.s009.DOC]

**S4 Table.** Information about existing hypertension prediction models developed using biomarkers (genetic risk score) from the selected studies

| **Study** | **Location Model Developed/**  **Ethnicity** | **Study Design** | **Age** | **Gender** | **Risk Factors Included** | **Events (n)/Total participants (N)** | **Definition of Outcome Predicted/Hypertension** | **Duration of Follow-up** | **Modeling Method** | **Discrimination** | **Calibration** | **Model Validation: internal or external** |
| --- | --- | --- | --- | --- | --- | --- | --- | --- | --- | --- | --- | --- |
| Yamakado et al.[60] 2015 | Japan/Asians | Prospective cohort | ≥ 20 years | Both male and female | PFAA Index 1: Leucine, alanine, tyrosine, asparagine, tryptophan, and glycine  PFAA Index 2: Isoleucine, alanine, tyrosine, phenylalanine, methionine, and histidine | 424/2637 | SBP ≥ 140 mm Hg or DBP ≥ 90 mm Hg or use of antihypertensive medication | 4 years | Logistic regression | NR | NR | Internal, leave-one-out cross-validation (LOOCV) and External, the independent validation dataset |
| Qi et al.[61] 2014 | China/Asians | Case-control | Case cohort: 64.48 ± 8.53 years; Control: 64.23 ± 10.13 years | Both male and female | rs17030613, rs16849225, rs1173766, rs11066280, rs35444, rs880315, rs16998073, rs11191548, rs17249754 | Patients: NR/1009, Controls = NR/756 | SBP ≥1 40 mm Hg or DBP ≥ 90 mm Hg or use of antihypertensive medication | NR | Logistic regression | NR | NR | NR |
| Lu et al.[62] 2015 | China/Asians | Prospective cohort | 35-74 years | Both male and female | Model1: GRS+ (Age, sex, and BMI) Model2: GRS +Model 1+smoking, drinking, pulse rate, and education  Model3: GRS+ Model2 + SBP and DBP | 2559/7724 | SBP ≥ 140 mm Hg or DBP ≥ 90 mm Hg or use of antihypertensive medication | Mean 7.9 years | Logistic regression and Cox proportional- hazards regression | Model1: C-statistic =0.650 [0.637-0.663] (without GRS), 0.655 [0.642-0.668] (with GRS) Model 2: C-statistic = 0.683 [0.670-0.695] (without GRS), 0.687 [0.675-0.700] (with GRS)  Model 3: C-statistic = 0.774 [0.763-0.785] (without GRS), 0.777 [0.766-0.787] (with GRS) | NR | NR |
| Zhang et al.[63] 2015 | China/Asians | Prospective cohort | 18-88 years | Both male and female | Five latent factors extracted from 11 biomarkers (BMI, SBP, DBP, FBG, TG, HDL-C, Hb, HCT, WBC, LC, NGC): inflammatory factor, blood viscidity factor, insulin resistance factor, blood pressure factor, and lipid resistance factor, and age | 3793/17,471 | SBP ≥140 mm Hg or DBP ≥90 mm Hg or use of antihypertensive medication | 5 years | Cox proportional- hazards regression | Derivation cohort: AUC = 0.755 [0.746-0.763] (men), AUC = 0.801 [0.792-0.810] (women) Validation cohort: AUC = 0.755 [0.746-0.763] (men), AUC = 0.800 [0.791-0.810] (women) | NR | Internal, 10-fold cross-validation |
| Zhao et al.[73] 2008 | China/Asians | Case-control | 35–74 years | Both male and female | MDR Model: 4-locus model consisted of the SNP KCNMB1-rs11739136, RGS2-rs34717272, PRKG1-rs1881597, and MYLK-rs36025624; CART Model: RGS2, PRKG1, KCNMB1, and MYLK genes | Total: 4759 (2411 hypertensive and 2348 age-matched and sex-matched healthy controls) | Average SBP ≥ 150 mm Hg, an average DBP ≥ 95 mm Hg, or current use of antihypertensive medication | NR | Multifactor-dimensionality reduction (MDR) and classification and regression trees (CART) | MDR Model: Accuracy = 52.98%, cross-validation consistency = 9.7 | NR | Internal, 10-fold cross-validation |
| Wang et al.[78] 2014 | China/Asians | Case-control | Average 64.48 ± 8.53 years (cases), 64.23 ± 10.13 years (control) | Both male and female | The best MDR model included rs5804 and BMI | 1009 hypertensive patients and 756 normotensive controls | Mean SBP ≥ 140 mmHg and/or DBP ≥ 90 mmHg on two occasions and/or the current usage of antihypertensive drug treatment | NR | Multifactor dimensionality reduction (MDR) model | The best MDR model testing accuracy = 0.6331, cross-validation consistency = 10 | NR | Internal, 10-fold cross-validation |
| Zhao et al.[74] 2014 | China/Asians | Case-control | Average 64.48 ± 8.53 years (cases), 64.23 ± 10.13 years (control) | Both male and female | The overall best model includes three-locus rs6749447, rs35929607, and rs3754777 | 1009 hypertensive patients and 756 normotensive controls | Mean SBP of at least 140 mm Hg or a mean DBP of at least 90 mm Hg or the current intake of antihypertensive drugs | NR | Multifactor dimensionality reduction (MDR) model | The best MDR model: testing accuracy of 0.7309 and a maximum cross-validation consistency of 10 (P < 0.001) | NR | Internal, 10-fold cross-validation |
| Niiranen et al.[54] 2016 | Finland/Whites | Prospective cohort | ≥ 30 years | Both male and female | Model 1: GRS  Model 2: Model 1 + age + sex  Model 3: Model 2 + smoking, diabetes, education, hypercholesterolemia, leisure-time exercise, and BMI | NR/2045 | BP ≥ 140/90 mm Hg and/or antihypertensive medication | 11 years | Multiple linear and logistic regression | C-index = 0.731 (Model 1) | NR | NR |
| Choi et al.[47] 2014 | USA/Mexicans | Prospective cohort | NR | Both male and female | Age, gender, smoke, age × gender, Rs10510257 (AA), Rs10510257 (AG), Rs1047115 (GT) | NR/443 | SBP >140 mm Hg, DBP >90 mm Hg, or use of antihypertensive medication | NR | Generalized estimating equations for Marginal model and logistic random effect model for Conditional model | Marginal model: AUC = 0.839 (with SNPs); Conditional model: AUC = 0.973 (with SNPs) | NR | NR |
| Lim et al.[48] 2015 | Korean/ Asians | Prospective cohort | 40-69 years | Both male and female | Traditional variables: age, gender, SBP, current smoking status, family history of hypertension, BMI, and one genetic variable (cGRS or wGRS derived from the 4 SNPs): rs995322, rs17249754, rs1378942, rs12945290 | NR/5632 | SBP ≥ 140 mm Hg or DBP ≥ 90 mm Hg or use of antihypertensive medication | 4 years | Logistic regression | Derivation cohort: C-statistic = 0.810 [0.796–0.824] (model without wGRS, C-statistic = 0.811 [0.797–0.825] (model with wGRS) Validation cohort: Mean C-statistic = 0.811 [0.809-0.816] | HL Chi-square = 6.916 (model without wGRS), HL Chi-square = 5.711 (model with wGRS) | Internal validation, fivefold cross-validation |
| Chien et al.[24] 2011 | Taiwan/Chinese | Prospective cohort | ≥ 35 years | Both male and female | Biochemical Model: Age, gender, BMI, SBP and DBP, white blood count, fasting glucose, uric acid | 1029/2506 | SBP ≥ 140 mmHg or DBP ≥ 90 mmHg or reported use of BP-lowering medications | Median 6.15 years | Weibull regression | Biochemical Model: AUC = 0.735 [0.715 - 0.755] (point based), AUC = 0.74 (coefficient based) | Biochemical Model: HL Chi-square = 13.2, p = 0.11 (point based), 6.4, p = 0.60 (coefficient based) | Internal, fivefold cross-validation |
