# Supplementary material for: Prediction of hypertension using traditional regression and machine learning models: A systematic review and meta-analysis
Source: PLoS One. 2022 Apr 7;17(4):e0266334. doi: 10.1371/journal.pone.0266334 (PMC8989291; doi:10.1371/journal.pone.0266334)
Supplement: Attachment — Submitted filename: Review.docx [file pone.0266334.s010.docx]

Review: Chowdhury et al “Prediction of hypertension using traditional regression and machine learning models: A systematic review and meta-analysis”

Overview

In this paper Chowdhury et al provide a systematic review and meta-analysis comparing prediction models for the development of hypertension in the general population derived using traditional regression-based and machine learning approaches.

Meta-analysis was only possible for measures of discrimination. Overall the pooled c-statistics on meta-analysis are similar and of moderate-good performance between traditional regression-based and machine learning derived models. High heterogeneity was found, with sources identified for traditional regression-based models through meta-regression. Only one model has been extensively externally validated (Framingham Hypertension risk model) but it showed significant heterogeneity in meta-analysis. Performance of risk models for hypertension have only been appropriately checked in Asian and Caucasian populations and clinical implementation has not been assessed.

Overall impression

I would like to congratulate the reviewers on an extremely thorough and methodologically sound systematic review and meta-analysis. My main concerns relate to the structure and writing of the discussion section, and the presentation the table.

Major issues

- The aims of the study are clearly delineated in the introduction (point 1-4). However I do not feel the structure of the discussion follows these aims or highlights the most salient findings of the analysis. Furthermore in my opinion the discussion section is too long. It would be better presented:
  - Major findings of the study (3-4 points)
  - Discussion of previous literature and how this differs
  - Future areas for research / gaps in knowledge
  - Limitations
  - Final conclusion
- The presentation of table 1 is extremely difficult to follow. The presentation of so many columns means that some of the entries for each study take up an entire page. It would be better to break this up into at least 2/3 tables e.g. between study population characteristics, model development characteristics/performance, variables used in model; and all these tables do not need to be in the main file (eg Himmelreich et al -> <https://academic.oup.com/europace/article/22/5/684/5721485>)
- Why are traditional regression model study characteristics included in main paper but not machine learning counterparts. It would be better to present them more equally
- There are wide prediction intervals suggesting significant heterogeneity. Have you considered a Bayesian approach for meta-analysis? Frequentist methods can produce prediction intervals with poor coverage when there is a mixture of study sizes (<https://pubmed.ncbi.nlm.nih.gov/30032705/>)

Minor issues

- I note some of the models for predicting hypertension use systolic blood pressure and diastolic blood pressure. Does this not appear ‘double-dipping’ to include a variable that may well be an outcome? Does this not require some comment?
- Page 13 line 330 – please be more specific than ‘basically’
- Page 15 line 388 – I belive it should be ‘models’
- Page 17 line 446-447 does not make sense
- Figure 1 – I believe the reasons for exclusion would be better ordered alphabetically or in descending number of records excluded
